# A Summary of the U.S. Marine Recruit Assessment Program (RAP) Procedures and Survey from 2003 to 2021

**Published:** 2024-02-20

**Authors:** Zeina G. Khodr, Jennifer McAnany, Yohannes G. Haile, Vanessa G. Perez, Patricia Rohrbeck

**Affiliations:** 1 Leidos, Inc.; 2 Military Population Health Directorate, Naval Health Research Center, San Diego, CA

## Abstract

The Recruit Assessment Program (RAP) is a cross-sectional, baseline survey of U.S. Marine recruits administered at Marine Corps Recruit Depot, San Diego. This report presents RAP study procedures and survey content that was administered to 229,015 participants between 2003 and 2021. Self-reported data were collected on recruit demographics, physical and mental health, adverse life experiences, lifestyle and risky behaviors, and substance use. In 2013, the survey was updated to remove questions with other linkable and reliable sources and those with low completion rates and low relevance to Marine health research; the removal of these items allowed for the addition of instrument measures for major depression, Post-Traumatic Stress Disorder, anger, and resilience with no significant change to overall survey length. Average completion rates are approximately 95%. Multiple studies have shown the utility of RAP data collected thus far as a robust data repository of pre-service health and behavioral measures.

**What are the new findings?:**

By surveying Marine recruits within days of arrival, RAP can more accurately obtain information on their lives and experiences prior to military service. RAP establishes a comprehensive profile of a recruit, creating a robust resource that takes into consideration each recruit’s unique history when assessing future experiences and concerns.

**What is the impact on readiness and force health protection?:**

The purpose of the RAP survey is to increase understanding of how pre-service exposures may affect a recruit’s health and readiness while in service, and thereby inform policy decisions that protect Marines’ mental and physical health while sustaining military readiness. RAP data can also be linked to other military data sources to assess how past experiences influence future decisions, behaviors, and outcomes that may affect operational health and readiness. Utilization of this additional level of information aids policy and intervention improvement efforts.

## 1 BACKGROUND

A major limitation in military health research is the inability to account for events that occurred prior to military service. A Presidential Review Directive,^1^ Institute of Medicine reports,^2^ and military advisory boards have emphasized the need for collection of baseline health data for pre-existing health conditions and risks.^3,4^ To further investigate concerns related to pre-service health and behavioral measures and military health and career progression, the Recruit Assessment Program (RAP) was developed by the Naval Health Research Center (NHRC).

RAP was implemented in June 2001 among male recruits at the Marine Corps Recruit Depot (MCRD), San Diego.^5^ While initially established with the goal of automating recruit enrollment within the electronic medical record system, RAP has evolved to focus on assembling a comprehensive profile of U.S. Marine recruits’ experiences prior to military duty.

The RAP survey consists of measures of Marine recruits’ pre-service health and behaviors not collected elsewhere. Collected during recruit training, RAP contributes to the understanding of a Marine recruit’s preservice mental, physical, social, and behavioral health, and it can inform prevention and intervention strategies for adverse outcomes such as sexual assault and military attrition.^6,7^ RAP provides baseline data for health risk assessments during general service in the Marine Corps, as well as those associated with operational deployments and within military occupational specialties. The ever-changing demographics of Marine recruit populations emphasize the need for self-reported, autonomous, baseline data to properly account for pre-service exposures when assessing the effects of military life.

Marine recruit training is a 13-week process, during which a recruit leaves civilian life and adapts to a Marine Corps lifestyle. Training takes place at MCRD, Parris Island, South Carolina, or at MCRD, San Diego, California. Thus far, RAP has collected data exclusively on recruits at MCRD San Diego, where each receiving company of 250 to 645 Marine recruits are offered the opportunity to complete a RAP survey.

This report summarizes survey administration and content for the RAP versions 4 and 5 (2003-2021), the predominant versions that provided 87.7% of all RAP data collected. The survey has gone through multiple revisions, with the first version in 2001 (n=17,424),^5^ versions 2 and 3 in 2002 (n=14,673),^8,9^ version 4 in 2003-2013 (n=134,761),^8^ followed by version 5, currently in use (n=94,254). Prior reports have provided detailed reviews of survey versions 1 through 3.^5,8,9,10^ Survey approval was obtained by the U.S. Marine Corps Survey Office. All data were collected voluntarily from participants who provided informed consent, with the approval of the Institutional Review Board at the NHRC.

## 2 METHODS

**Survey Content**

Survey content was developed utilizing standardized survey instruments in combination with subject matter expert (SME) and stakeholder recommendations. Approximately 32% of the content in version 4 was removed from version 5, with new content in version 5 comprising 13% (**Table 1**). Removal of version 4 questions that had other linkable and reliable sources, low completion rates, or low relevance to Marine health stakeholders allowed the addition of instrument measures for major depression, Post-Traumatic Stress Disorder (PTSD), anger, and resilience.

**Demographics**

Demographic data collected include date of birth (DoB), most recent home location, race and ethnicity, education, marital status, handedness, height, and current and past year weights. Family demographic data include familial structure, education, and ability to provide essential needs. Military demographic data collected include prior service, reason for joining, and parental military service.

**Health**

*Physical Health*

Physical health questions were adapted from the Seabee Survey of Health Conditions,^11^ the 12-Item Short Form Survey (SF-12),^12^ and the 36-Item Short Form Survey (SF-36).^13^ Specific health symptoms surveyed, ranging from chronic cough to muscle aches, were reduced from 23 to 16 questions in versions 4 and 5, respectively.

*Mental Health*

Version 4 included SF-36 questions about reasoning and problem-solving, forgetfulness, attention, and concentration within the past year.^13^ Version 5 included the following validated instrument measures: the 8-item Patient Health Questionnaire (PHQ-8) depression scale^14^; the Primary Care PTSD Screen for DSM-5 (Diagnostic and Statistical Manual of Mental Disorders, Fifth Edition) (PC-PTSD-5)^15^; the 7-item Pearlin Mastery Scale^16^; and 1 question assessing self-perception of resilience.^17^

*Medical Care*

Version 4, but not version 5, included questions about medical care during the past 5 years such as care received, reasons for recommended preventive care (e.g., medical or dental checkup), hospitalizations, and prescription use.

**Life Experiences**

*Lifestyle and Risky Behaviors*

Multiple questions focus on lifestyle habits, as well as risky behaviors, including diet, sleep, television, schooling, driving safety, sexual activity, emotional behavior, social support, and religious activities. Age at first sexual intercourse, condom use, and sexually transmitted infection (STI) diagnosis were ascertained in both versions. Questions about anger were expanded to include the Dimensions of Anger Reactions (DAR-5) scale.^18,19^

*Adverse Experiences*

Ten standardized questions on adverse childhood experiences (ACEs) were included in the survey,^20^ in addition to questions about adverse experiences throughout life as well as in the past year (e.g., self-harm, attacked, raped, arrested, etc.). Questions about attention deficit hyperactivity disorder (ADHD) were reduced in version 5. Three questions on traumatic brain injury (TBI), adapted from the Post Deployment Health Assessment (PDHA), were added in version 5.^21^ Past occupational exposures were collected for participants who had worked in a position for more than 1 month.

*Substance Use*

Questions about tobacco use were adapted from prior military health surveys such as the Seabee Health Study^11^ and the National Health Survey of Persian Gulf War Era Veterans.^22^ Questions specific to cigarettes focused on age at first use, cessation attempts, last use, and typical use. Use of pipes, hookahs, cigars, and smokeless tobacco products were also surveyed. Detailed questions about alcohol use, age at first use, frequency, quantity, and behavior were included with the Cut, Annoyed, Guilty, and Eye (CAGE)^23^ instrument and 3-item Alcohol Use Disorders Identification Test (AUDIT)-C.^24^ Version 5 also included the full 10-item AUDIT.^25^

**Survey Administration**

RAP was designed to be a self-reported, paper and pencil-based survey that could be completed within 45 minutes, administered in various induction settings, and generalizable to a culturally diverse population.^9^ Once a week, Drill Instructors (DIs) led receiving recruits into an auditorium-style classroom, where blank RAP survey booklets were placed at each seat.

To allow for autonomy, DIs were not present during the survey’s introduction, when recruits were provided an approximately 15-minute verbal briefing on RAP and their option for voluntary participation. NHRC researchers presented slides reviewing the survey’s “Consent to Participate in Research” section, addressing: 1) voluntary participation, 2) benefits and risks, 3) data confidentiality and security, and 4) if desired, guidance for study withdrawal. Version 5 included options for consent to use of the data in future research beyond the RAP protocol purview, along with a Health Insurance Portability and Accountability Act (HIPAA) authorization for permission to link to protected health information (PHI).

Following each briefing, recruits were given time to independently review the consent forms and discuss questions or concerns with the research team. Once the allotted hour for survey completion passed, recruits returned their surveys and were dismissed to their DIs. All recruits returned their survey, whether completed or not.

**Survey Processing**

Surveys were processed at NHRC. Surveys without consent or with consent withdrawn were destroyed. Surveys with consent were scanned using Teleform software (OpenText Corporation, Waterloo, Ontario, Canada) and entered into the RAP SQL database. A sample of 3-5% of surveys were manually checked for accuracy.

SAS version 9.4 (SAS Institute, Cary, NC) was used to manage and prepare the final analytic RAP database. Self-reported identifiers (e.g., Social Security number, date of birth, name) were verified with Defense Manpower Data Center (DMDC) records and entered if necessary. Implausible outliers for variables such as age, height, and weight were set to ‘missing.’ Survey versions were cleaned separately, prior to consolidation in a final analytic dataset, as they differed in question order, response options, skip patterns, and study domains. Questions from different survey versions were standardized when possible, with raw variables retained.

Historically, participation rates were calculated from denominators generated from DMDC records, which were prone to misclassification. In 2012, participation rate accuracy was improved by documenting the total numbers of recruits present.

## 3 RESULTS

**Participants**

From 2003 through 2021, 229,015 recruits completed the survey; 134,761 participants completed version 4 (2003-2013) and 94,254 completed version 5 (2013-2021). DMDC demographic data were utilized to verify 99.6% of participants as Marine service members. Peaks in enrollment count mirrored peak recruitment periods, with highest participation in June. The average number of participants enrolled was approximately 12,000 recruits per year, with a high of 17,198 (7.51%) enrolled in 2005 and a low of 5,945 (2.60%) in 2020, when survey administration was paused from April through August due to the COVID-19 pandemic. As MCRD San Diego exclusively enrolled male Marine recruits until March 2021, RAP version 5 was the first to include female participants (n=109).

**Completion Rates**

**Figure 1:**
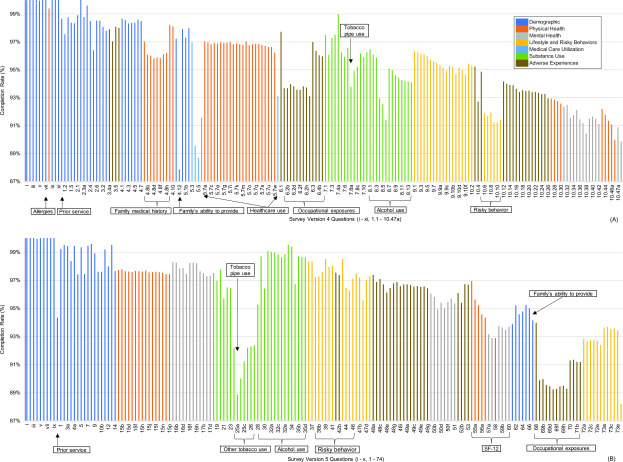
Completion Rates for 2003-2013 RAP Survey (A) Version 4 (n=134,761 Participants) and 2013-2021 RAP Survey (B) Version 5 (n=94,254 participants)

Average participation rates were 88.1% between 2012 and 2021, with a high of 97.8% in 2017, which decreased in 2020 and 2021 to 74.4% and 69.3%, respectively, due to implementation of guidelines for COVID-19 pandemic restrictions on movement. Average completion rates for individual survey questions were 94.5% and 95.3% for versions 4 and 5, respectively, and accounted for skip questions (**Figure 1**). The minimum completion rate was approximately 87% for both surveys. Completion rates for the final question declined to 89.9% and 88.2% for the final questions of versions 4 and 5, respectively.

Lower completion rates were observed in both surveys for questions not applicable to most participants (e.g., SF-12 questions about general health; use of tobacco products other than cigarettes, such as pipes; past occupational eposures). Marine recruits are a young and healthy population less likely to suffer from physical health issues that limit daily activities. Approximately 28% of participants reported themselves to be smokers, with a 97% completion rate in version 4 for smoking habit questions, and only 94% for pipe and cigar questions, which declined to 96% and 89%, respectively, in version 5. Completion rates were lower for questions discussing sensitive topic areas, although some improved after survey question reordering, specifically familial ability to provide essential needs (87.9%, version 4; 94.1%, version 5), family medical history (95.8-98.2%, version 4), prior health care provision (88.7-89.5%, version 4), alcohol use (91.4-96.5%, version 4; 95.3-99.5%, version 5), and risky behaviors (91.2-94.9%, version 4; 96.3-98.5%, version 5).

**RAP Publications**

RAP has demonstrated its capacity as a cross-sectional, individual resource^26,27^ that is also readily linkable to other data sources for longitudinal assessment of military health research (**Table 2**).^6,7,26,28,29,30,31,32,33,34,35,36^ Past RAP studies have successfully linked with data from the DMDC,^6,26,33^ PDHAs,^28^ the Naval Criminal Investigative Service Consolidated Law Enforcement Operations Center,^7^ and Department of Defense Medical Mortality Registry^33^; as well as other self-reported epidemiological data such as the RAP II,^30,34^ a follow-up survey for deployed RAP participants, and the Millennium Cohort Study, the largest self-reported, longitudinal military cohort study.^36^ More importantly, the study domains included in RAP surveys have demonstrated relevancy to military outcomes of interest, through findings from prior literature.

## 4 DISCUSSION

RAP data are proven to complement personnel data, which may not be as candid due to their collection during assessment for military duty. RAP data should be evaluated within the context of certain limitations. Currently, RAP administration is limited to MCRD San Diego and does not represent recruitment for the eastern U.S. Thus far, research studies using RAP data are limited to male participants, as MCRD San Diego did not include female recruits until 2021. Inherent limitations of self-reported, paper-and-pencil survey studies include low participation rates, misreporting of sensitive topics, and resource-intensive administration. Lastly, the lengthy RAP survey is administered during a strenuous time in a recruit’s career, which can increase respondent fatigue. Although question completion rates decreased nearer the survey’s end, they remained high, however, at 87%.

SME and stakeholder reviews have guided survey revisions that will allow future research on pre-service mental health and psychosocial factors as predictors for retention, mental health care provision, and suicide ideation and completion. Ongoing survey content considerations include media use, marijuana use, diversity and inclusion, sexual orientation, and women’s reproductive health. The ever-changing demographics of Marine recruit populations emphasize the need for resources such as RAP, which continues to evolve with changing needs for military operational health and readiness.

## Figures and Tables

**Table 1:** Changes to RAP Survey Questions by Version, Excluding Minor Edits to Questions, Prompts, or Response Options

**Survey Question Topic**		**Version 4 (2003-2013)**	**Version 5 (2013-2021)** (peak of ivermectin dispensation)
**Demographics**	State and country of birth	Included	Removed
	Size of hometown	Included	Removed
	Twin status	Included	Removed
	Parental military service	Included (yes/no response)	Added question on number of years in service
	Race and ethnicity	Included as a combined question	Separated race ('unknown' response) and ethnicity question
	Marital status	Included ('living together' response)	Separated questions about current and past marital status
	Weight in last year	Included (multiple choice response)	Changed (3-digit numeric field response)
	Parental death	Included ('before/after age of 10' response)	Change (yes/no response)
	Number of siblings	Included (2-digit numeric field response)	Changed (multiple choice response)
	Maternal and paternal education	Included as 2 separate questions	Combined as 1 question
			
**Health**	Health issues: acne, sleepwalking, bed wetting, stuttering, frequent indigestion, constipation or loose bowels, and pain or problems during sexual intercourse	Included	Removed
	Allergies	Included	Removed
	Family history of health conditions	Included	Removed
	SF-36 questions about changes in health over the past year	Included 7 additional questions	Removed 7 questions
	Medical care	Included	Removed
	Health questions on memory and critical thinking	Included 4 additional questions	Removed 4 questions
	SF-12 questions on physical and emotional health	Included (yes/no response)	Changed (Likert scale response)
	PHQ-8	Not included	Added
	PC-PTSD-5	Not included	Added
	7-item Pearlin Mastery Scale	Not included	Added
	Self-perception of reilience	Not included	Added
			
**Life experiences**	ADHD, hyperactivity, or learning disability treatment	Included	Removed
	Number of years ever home-schooled	Included	Removed
	Ever taking diet pills, laxatives, or vomiting for weight loss	Included	Removed
	Have children in the last year	Included	Removed
	Parental divorce	Included ('before/after age of 10' response)	Changed (yes/no response and 'separated/divorced')
	TBI	Not included	Added
	Household member imprisoned	Not included	Added
	DAR-5 scale	Not included	Added
	Ever spending night in jail	Not included	Added
			
**Substance use**	Likert scale question on smoking cigarettes in the last year	Included	Removed
	Alcohol use in the last year, total years ever, or while driving	Included	Removed
	Skip patterns in tobacco use section and collapsed response options	Not included	Added
	Skip patterns in alcohol use section	Not included	Added
	10-item full AUDIT	Not included	Added

Abbreviations: RAP, Recruitment Assessment Program; PHQ-8, 8-Item Patient Health Questionnaire depression scale; PC-PTSD, Primary Care Post-Traumatic Stress Disorder Screen for DSM-5; TBI, Traumatic brain injury; DAR, Dimensions of Anger Reactions; AUDIT, Alcohol Use Disorders Identification Test; ADHD, Attention-deficit/hyperactivity disorder; SF, Short Form Survey.

**Table 2:** RAP Publications Linking RAP Survey Data with Other Data Sources for Epidemiological Study

**First Author**	**Publication Year**	**Data Source(s)**	**Findings**
Larson [28]	2009	DMDC, PDHA, and MDR	Post-deployment psychiatric disorders were associated with low pay grade, hospitalization during deployment, low education, pre-service smoking, and PTSD symptoms at end of deployment. Findings supported expansion of combat-related questions in the PDHA.
Leardmann [29]	2010	DMDC and MDR	Childhood physical neglect was associated with post-deployment PTSD. Findings suggest Marines who experience multiple types of ACEs have increased risk for post-deployment PTSD.
Phillips [30]	2010	DMDC and RAP II	Risk factors for post-deployment PTSD included pre-service measures of violence exposure and combat experiences of feeling in great danger of death, being shot or seriously injured, and witnessing someone wounded or killed. Follow-up measures of military rank, social support, and number of deployments were also associated with post-deployment PTSD.
Booth-Kewley [31]	2010	DMDC and MDR	The strongest predictors of bad conduct discharges and military demotions among combat-deployed Marines were post-combat psychiatric diagnoses and younger age. These results indicate that combat-related psychological disorders may manifest as impulsive, disruptive, and antisocial behavior.
Horton [26]	2014	Physical fitness of recruit and DMDC	Trends in pre-service health and behavioral measures during the OEF/OIF era were improvements in BMI and physical activity levels and increases in smokeless tobacco use, caffeine use, and angry outbursts.
Feinberg [32]	2015	Physical fitness of recruit	Smoking cessation in this cohort of male, Marine recruits resulted in improved physical aerobic performance, independent of other pre-service health and behavioral measures. Average recruit running speeds improved among all recruits, but improvement was greater among prior smokers compared to recruits with no history of smoking.
White [6]	2016	DMDC	Pre-service risk factors for military attrition due to drug use included endorsement of a Black racial group, incomplete high school education, joining the military to "leave problems at home," and an arrest within the year prior to joining the military. Attrition due to drug use accounted for a significant loss in service years of trained service members.
Phillips [33]	2017	DOD Medical Mortality Registry, DMDC, and MDR	Risk factors for suicide completion included incomplete high school education and current smoking behavior at enlistment. Diagnoses of TBI or depression and relationship counseling during military service also were risk factors for suicide completion. Suicide prevention efforts should not preferentially focus on deployed service members, as deployment was not an independent risk factor.
Bauer [34]	2020	RAP II	Deployed Marines were less likely to reduce fast food consumption versus non-deployed Marines at 3 years of follow-up from enlistment. Combat-deployed Marines, compared with non-deployed Marines, had increased odds of several adverse health-related behaviors post-deployment including binge drinking, new-onset alcohol dependence, initiation of smoking, and reduced seat belt use.
Leardmann [7]	2022	NCIS	A low prevalence of sexual offenses over a 9-year period were observed (0.01%). Pre-service health and behavioral measures associated with sexual misconduct while in military service included endorsement of American Indian/Alaskan Native, Hispanic, and Multiracial/Other race and ethnicity categories; incomplete high school education; adverse experiences such as parental death and school suspension/expulsion; and unprotected sex.
Reed-Fitzke [36]	2023	MCS	Subgroups of exposure to pre-service ACEs indicative of adversity, such as parental absence, have more impact on risk for depression and PTSD symptoms versus absolute count of ACEs. These associations were modified by combat exposure when assessing moderate adversity with parental loss.
MacGregor [37]	in preparation	PHA	New-onset alcohol misuse was more likely among Marines who turned 21 years old during follow-up. Pre-service experiences associated with alcohol misuse in this study included a higher ACE score, job dismissal, and witnessing a stranger injured or killed.

Abbreviations: RAP, Recruit Assessment Program; PDHA, Post Deployment Health Assessment; MDR, Military Health System Data Repository; PTSD, Post-traumatic stress disorder; ACE, adverse childhood experience; OEF / OIF, Operation Enduring Freedom/Operation Iraqi Freedom; BMI, body mass index; DOD, Department of Defense; TBI, traumatic brain injury; NCIS, Naval Criminal Investigative Service; MCS, Millennium Cohort Study; PHA, Periodic Health Assessment.

## AUTHOR AFFILIATIONS

Military Population Health Directorate, Naval Health Research Center, San Diego, CA: Lt Col Rohrbeck; Leidos, Inc.: Dr. Khodr, Dr. McAnany, Mr. Haile, Ms. Perez

## DISCLAIMERS

Lt Col Rohrbeck is a military service member. This work was prepared as part of official duties. Title 17, U.S.C. §105 provides that copyright protection under this title is not available for any work of the U.S. Government. Title 17, U.S.C. §101 defines a U.S. Government work as work prepared by a military service member or employee of the U.S. Government as part of that person’s official duties. Report 23-31 was supported by the Military Operational Medicine Research Program under work unit 60002.

This study protocol was reviewed and approved by the Institutional Review Board of the Naval Health Research Center, in compliance with all applicable Federal regulations governing human subject participant protection. Research data were derived from an approved Naval Health Research Center Institutional Review Board protocol NHRC.2000.0003.
